# Supplementary material for: A 12-Week, Randomized, Double-Blind, Placebo-Controlled Study to Evaluate the Efficacy and Safety of Lactobacillus plantarum LMT1-48 on Body Fat Loss
Source: Nutrients. 2025 Mar 28;17(7):1191. doi: 10.3390/nu17071191 (PMC11990557; doi:10.3390/nu17071191)
Supplement: Supplementary file 1 [file nutrients-17-01191-s001.zip › nutrients-3537159-supplementary.pdf]

Supplementary Materials

Figure S1. Clinical trial timeline

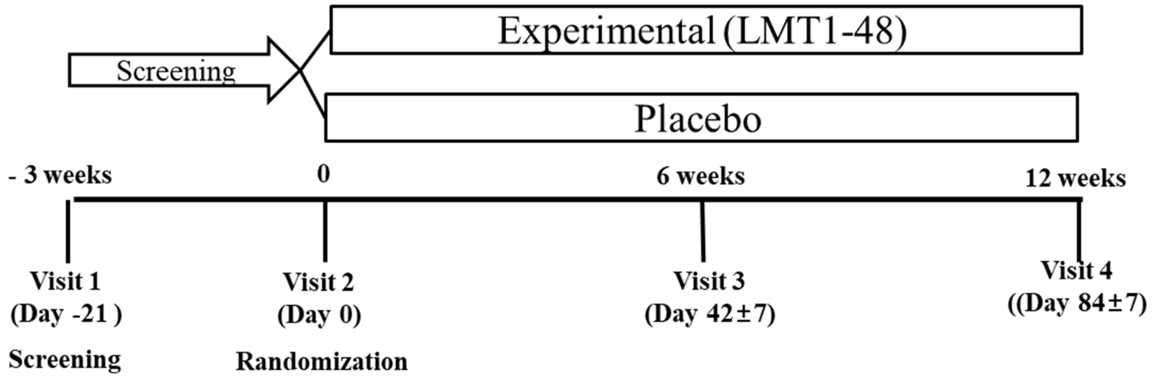

Figure S2. Flow diagram of subject enrollment, allocation, follow-up and analysis

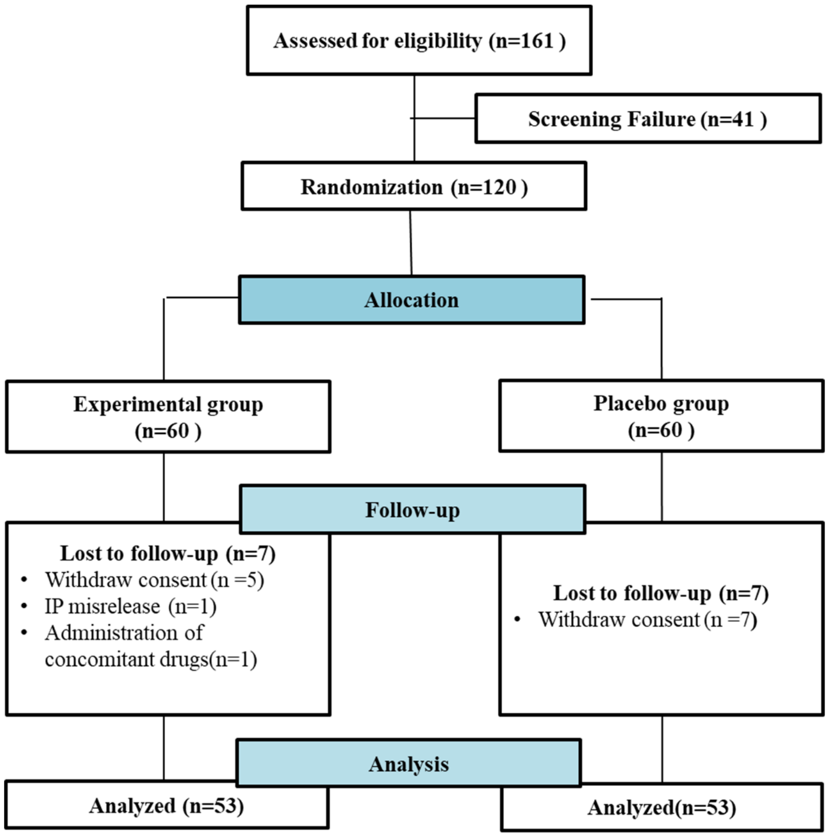

Table S1. Summary of sequencing information

Group	Placebo		Experimental		Total
	Baseline	Week 12	Baseline	Week 12	
Number of samples	53	53	53	53	212
Total number of seqs	1,224,505	1,193,622	1,192,982	1,251,535	4,862,644
Average number of seqs ± SD	23,104 ± 7869	22,521 ± 6935	22,509 ± 8105	23,614 ± 6989	22,937 ± 7453
Total number of features	2636	2620	2357	2306	5410
Average number of features ± SD	167 ± 50	166 ± 48	164 ± 51	161 ± 46	165 ± 49

Table S2. The Bacteriodetes to Firmicutes ratio

Ratio	Placebo	Experimental
Baseline	0.697 ± 0.983	0.627 ± 0.475
Week 12	0.499 ± 0.437	0.600 ± 0.429
Change from baseline		
p-value (1)	0.124	0.730
p-value (2)	0.097	-

Table S3. Summary of adverse events

	Placebo (*n* = 60)	Experimental (*n* = 60)	p - value
Total adverse events (*n*, %)	7 (11.7)	5 (8.3)	0.543
Gastrointestinal disorders (*n*, %)	1 (1.7)	2 (3.3)	1.000
Skin tissue disorders (*n*, %)	1 (1.7)	0 (0.0)	1.000
Musculoskeletal disorders (*n*, %)	1 (1.7)	0 (0.0)	1.000
Nervous system disorders (*n*, %)	0 (0.0)	1 (1.7)	1.000
Reproductive disorders (*n*, %)	2 (3.3)	0 (0.0)	0.496
Infections (*n*, %)	2 (3.3)	2 (3.3)	1.000
Serious adverse events (*n*, %)	0 (0.0)	0 (0.0)	-

Table S4. Blood anaylses for safety

	Group	Baseline	Week 12	p-value†	p-value‡
AST (IU/L)	LMT1-48	27.4 ± 12.4	25.5 ± 13.6	0.220	0.706
	placebo	24.3 ± 7.2	23.0 ± 6.7	0.259	
ALT (IU/L)	LMT1-48	27.8 ± 17.9	25.9 ± 19.3	0.465	0.857
	placebo	26.8 ± 20.1	22.7 ± 13.5	0.083	
BUN (mg/dL)	LMT1-48	13.1 ± 3.5	13.2 ± 3.5	0.848	0.883
	placebo	12.9 ± 2.9	12.8 ± 3.0	1.000	
Creatinine (mg/dL)	LMT1-48	0.75 ± 0.18	0.74 ± 0.17	0.431	0.535
	placebo	0.74 ± 0.13	0.74 ± 0.14	0.949	
WBC (10^3/μL)	LMT1-48	6.29 ± 1.54	6.24 ± 1.65	0.703	0.563
	placebo	6.47 ± 1.60	6.50 ± 1.81	0.313	

† Compared within groups; ‡ Compared between groups
